# Unlocking potential: Development finance reforms needed to support localized health product manufacturing

**DOI:** 10.1371/journal.pgph.0005369

**Published:** 2026-01-08

**Authors:** Sharonann Lynch, Ifedayo Adetifa, Jicui Dong, Robert Matiru, Cassidy Parshall

**Affiliations:** 1 Center for Global Health Policy and Politics, Georgetown University, Washington, DC, United States of America; 2 Foundation for Innovative New Diagnostics (FIND), Geneva, Switzerland; 3 Local Production and Assistance Unit, Innovation and Emerging Technologies Department, Access to Medicines and Health Products Division, World Health Organization, Geneva, Switzerland; 4 Programme Division & Regional Manufacturing, Unitaid, Geneva, Switzerland; PLOS: Public Library of Science, UNITED STATES OF AMERICA

While nearly half of the world lacks access to diagnostics [1], the world’s medicines and diagnostics are produced by a narrow set of global manufacturers, concentrated mostly in high-income countries. Except for a few key countries, low- and middle-income countries (LMICs) have historically played a limited role in manufacturing the health products needed for resilient health systems, contributing to pronounced inequity in access. In Africa over 70% of pharmaceuticals and 95% of active pharmaceutical ingredients are imported [2] and only 4% of diagnostics in Latin America were produced locally during the COVID-19 pandemic [3].

Communities worldwide face additional barriers to essential medicines and diagnostics due to United States foreign aid cuts [4]. Meanwhile, France, Germany, and the United Kingdom are reducing official development assistance by 9–17% in 2025 [5]. Consequently, multilateral development organizations are struggling to maintain their full operations amidst the large vacuum in global health funding [6]. Dependable access to health products and services for many communities remains precarious, with global funding cuts estimated to result in almost three million additional HIV-related deaths between 2025 and 2030, threatening decades of progress for the global HIV response [7].

While there has been growing political momentum towards localizing health product manufacturing, current challenges facing the global health ecosystem have magnified the need for urgent action to secure sustainable access to health products across LMICs. Local manufacturing in LMICs can also help diversify supply chains and enable a more effective global response during future outbreaks. As international health funding drops to a 15-year low [8], development finance institutions (DFIs) across both high-income countries and LMICs have the opportunity and the imperative to step up to support long-term health security, adopting new strategies to increase the capital provided for scaling-up localized manufacturing.

## Financing is a critical enabler for localized manufacturing

Financing is a critical enabler for local and regional manufacturers to scale-up manufacturing capacity, invest in quality assurance, and diversify product production. Across LMICs, global health actors are providing key support for vaccine manufacturers [9]; however, projects targeting the manufacturing of medicines and diagnostics are limited and could face increasing uncertainty. In March 2025, the African Development Bank announced the approval of a EUR 15 million loan for an Egyptian manufacturer to strengthen the supply of biosimilars for countries across Africa and the Middle East [10]. The Asian Development Bank is financing the production of diagnostics kits in Bangladesh [11], and in Latin America and the Caribbean, while the Inter-American Development Bank (IDB) is supporting 14 pharmaceutical manufacturing projects across eight countries [12].

**Fig 1:**
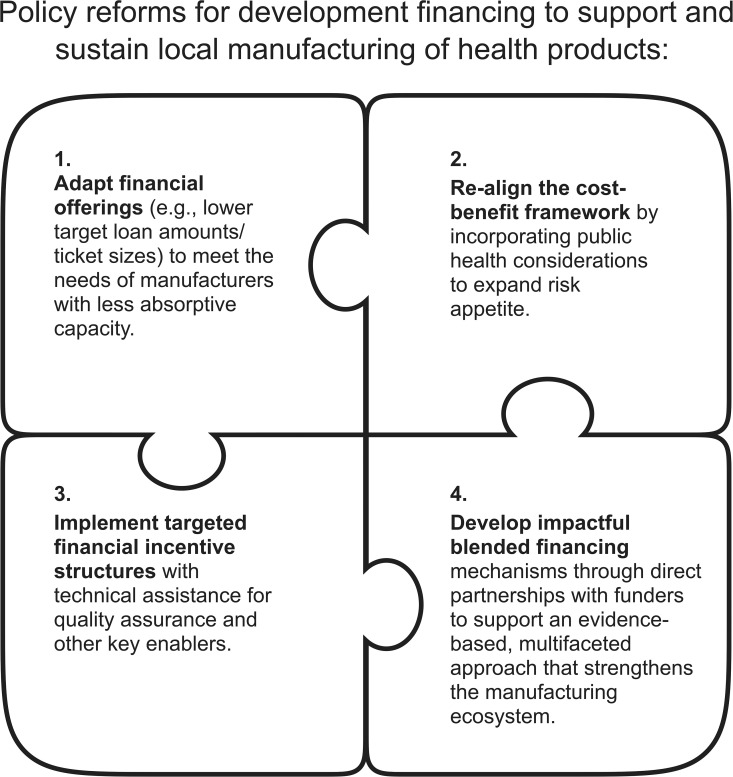
Policy reforms for development financing to support and sustain local manufacturing of health products.

Despite this progress, there remains significant untapped potential. To accelerate the development of manufacturing capacity in LMICs, DFIs should adopt new and innovative strategies that are well-suited for the pharmaceutical and diagnostic sectors while leveraging partnerships across the global health ecosystem (Fig 1).

## Strategies for development finance institutions to spur and sustain local health product manufacturing

The current business model and financial offerings of DFIs must adapt to meet the needs of health product manufacturers. Small- to medium-sized manufacturers in LMICs often need operational upgrades and technical assistance to develop and sustain quality systems while also scaling up production, requiring significant capital investment and collaboration among partners. However, these manufacturers may be ineligible for DFI financing due to stringent “bankability” criteria, including minimum loan sizes that exceed local manufacturers’ capacity. Few LMIC manufacturers—even those well-positioned to support local health needs—receive financing as a result. Reducing minimum loan requirements would enable more qualifying projects and increase overall DFI financing.

Diagnostics provide a particularly strong use case for DFIs. Compared to many vaccines or biologics, the product development cycle for diagnostics is shorter, less complex, and requires comparatively less investment. At the same time, early-stage diagnostic companies face long timelines before reaching procurement eligibility. During this period, they must maintain operations and quality systems without steady revenue, creating a financing gap. DFIs should therefore consider instruments such as flexible working capital or blended financing to help manufacturers bridge this critical period and avoid collapse before market entry.

The current investment model may also be missing the true value of localized production, resulting in a more limited risk appetite. Opportunities to gain traction through lower-risk investments are important, but the cost-benefit framework must adapt to incorporate the full suite of benefits that are afforded by increased manufacturing capacity, such as health security and resilient global supply chains.

Further, increasing in-house pharmaceutical and diagnostic expertise within DFIs can contribute towards a higher risk tolerance while enabling them to better leverage targeted incentives within their investment framework. While DFIs traditionally rely on external sources for technical expertise within the health product manufacturing space, further developing this in-house capacity will strengthen their ability to strategically deploy capital to promote the achievement of key enablers that are essential for manufacturers to successfully sustain production capacity, such as quality assurance.

## Leveraging partnerships to transform the health product manufacturing ecosystem

Beyond access to affordable capital, creating a supportive ecosystem is critical for sustainable manufacturing capacity. From skilled workforce development and regulatory harmonization to securing offtake agreements and demand generation, DFIs should play a larger role in ecosystem strengthening, collaborating with other key actors such as national governments, regulatory bodies, and procurement agencies to overcome barriers that would hinder the successful scale-up of manufacturing. One such collaboration between the World Health Organization (WHO), Unitaid, the Global Fund to Fight AIDS, Tuberculosis and Malaria (the Global Fund), International Finance Corporation, the Africa Centre for Disease Control and Prevention (Africa CDC), and other partners is working towards regional manufacturing and procurement capacity in Africa. In May 2025, the Global Fund announced that an HIV treatment manufactured by a Kenyan pharmaceutical company, and supported by Unitaid to achieve WHO prequalification, is being procured for use in Mozambique – a historic milestone for the continent [13].

As financing is deployed, DFIs could closely coordinate with technical partners such as WHO’s Local Production and Assistance Unit to ensure that manufacturers have the tools and technical assistance needed to achieve sustainable and quality assured production. Additionally, demand generation is central to a sustainable manufacturing ecosystem, and DFIs must consider innovative ways to secure demand for locally manufactured health products [14]. For example, Unitaid and partners have developed a financing mechanism to expand medical oxygen access in East Africa that combines catalytic financing with volume guarantees [15]. Greater coordination among DFIs could also support more effective mobilization of financing.

At a time when global health funding is contracting and inequities in health product access are pronounced, DFIs can take meaningful steps towards addressing longstanding challenges in scaling up local manufacturing capacity for health products. With targeted reforms and strategies, DFIs are well-positioned to support localized manufacturing to address current health challenges and provide greater security against future risks. Building a health product manufacturing ecosystem that centers equity and justice must be prioritized for immediate action as we work towards a new paradigm that realizes global access, health security, and system resilience.
